# Awareness and Knowledge of Adverse Drug Reactions and Pharmacovigilance Among Medical and Nursing Students and Staff in a Tertiary Care Hospital

**DOI:** 10.7759/cureus.69981

**Published:** 2024-09-23

**Authors:** Shrinivas R Raikar, Sneha Sneha, Sreeraj G, Janarthanan R

**Affiliations:** 1 Pharmacology, Bijapur Lingayat District Educational University (BLDE Deemed to be University) Shri B M Patil Medical College Hospital and Research Centre, Vijayapura, IND

**Keywords:** adverse drug reaction, awareness, central drugs standard control organization, indian pharmacopoeia commission, knowledge, national coordination center, pharmacovigilance, uppsala monitoring centre, world health organization

## Abstract

Background

Pharmacovigilance is defined as the science and activities related to the detection, assessment, understanding, and prevention of adverse effects or other drug-related problems. Underreporting of adverse drug reactions (ADRs) is a global issue, and adequate knowledge among healthcare professionals is essential. Engaging young doctors and nursing professionals in pharmacovigilance is crucial for promoting ADR reporting.

Objectives

The objective of this study is to evaluate the knowledge and awareness of ADRs and pharmacovigilance among medical and nursing students and staff in a tertiary care hospital in Vijayapura, India.

Methods

A cross-sectional, observational, questionnaire-based study was carried out using a validated questionnaire that included a total of 19 questions related to knowledge and awareness aspects. This was distributed electronically using Google Forms. By using the convenience sampling method, a sample size of 96 participants was calculated for each group (medical and nursing), and this was rounded to 100 participants per group to account for potential dropouts and to ensure the strength of this study. All the questions were peer-reviewed by expert faculties from the Department of Pharmacology.

Results

The data analysis showed that medical students and staff had superior knowledge of ADRs and pharmacovigilance. Nursing students and staff exhibited greater awareness of the nearest pharmacovigilance center and ADR monitoring centers in India. Nursing students and staff were more active in ADR and pharmacovigilance awareness programs.

Conclusions

This study clearly shows the importance of flexible attitudes and robust education in pharmacovigilance and ADR reporting among healthcare professionals. Executing simulation-based training can strengthen the skills and confidence of both medical and nursing professionals in ADR reporting and pharmacovigilance.

## Introduction

Drug disasters have heightened the importance of understanding and monitoring adverse drug reactions (ADR). The thalidomide catastrophe highlighted the critical need for robust drug safety regulations. It also initiated the global implementation of spontaneous ADR reporting systems [1]. The WHO defines an ADR as any noxious and unintended response to a drug occurring at doses used for prophylaxis, diagnosis, or therapy in humans, excluding failure to achieve the intended purpose [2].

Pharmacologically, a drug can elicit three types of effects: the therapeutic effect, the adverse effect, and the idiosyncratic effect. Therefore, it is essential to monitor both known and unknown adverse effects of drugs. This concept was often summarized in ancient times. Paracelsus, who is recognized for laying the foundation of modern toxicology, was the first to express the opinion that the most important factor to define the toxicity of a substance is its dose. The famous quote said by him is “All things are poison, and nothing is without poison; it is only the dose that determines whether something is not a poison” [3]. Several studies identified ADR as a major cause of morbidity and mortality [4]. Indiscriminate use of drugs, the constant influx of new drugs into the market, and the lack of an ADR reporting culture have led to an increase in the incidence of ADR [5]. Serious ADR accounts for 6-7% of all hospital admissions [6]. It is the moral duty of doctors and other healthcare professionals to provide information on suspected ADR, which is a part of patient care [7]. Spontaneous ADR reporting is crucial for monitoring both known and unknown adverse effects of drugs. This practice has been instrumental in detecting unusual and serious ADRs during the post-marketing phase, leading to the withdrawal of various drugs in the past like rofecoxib, cisapride, terfenadine, etc. [8]. The prevention of unnecessary hospitalizations due to ADRs is a key objective in health policy. Thus, in a country like India, with its vast population and diversity, it is essential to establish a standardized pharmacovigilance program in all medical colleges and hospitals across the nation.

The Pharmacovigilance Programme of India (PvPI), launched in July 2010 by the Central Drugs Standard Control Organization under the Ministry of Health and Family Welfare, was initially coordinated by the All India Institute of Medical Sciences, New Delhi, to improve patient safety for more than a billion people in India. In April 2011, the National Coordinating Centre was shifted to the Indian Pharmacopoeia Commission (IPC) in Ghaziabad, India, which now operates under the Uppsala Monitoring Centre-WHO (UMC-WHO). The program aims to detect substandard medications, prescribing patterns, and administration errors. The UMC-WHO maintains an international ADR database, collecting reports from national centers worldwide. The National Coordination Centre-Pharmacovigilance Program of India, IPC, and the Ministry of Health and Family Welfare, Government of India, were launched as a WHO Collaborating Centre for Regulatory Services on October 30, 2017. At present, there are 976 ADR monitoring centers that are actively functioning in India under the PvPI [9]. Pharmacovigilance involves the ongoing assessment and enhancement of drug safety. This regular evaluation is important, as many adverse effects can be managed or reversed through dosage adjustments or stopping the medication. In a country like India, an effective pharmacovigilance program is necessary for safeguarding vulnerable populations with varying cultural practices, disease patterns, socioeconomic factors, and medical systems [10].

Encouraging a culture of ADR reporting among the healthcare professionals who are closely involved in patient care is critical. The systematic data collection and analysis on ADR will not only help in safeguarding patients but also contribute to the improvement of therapeutic outcomes and overall healthcare practices.

## Materials and methods

Study design

This is a cross-sectional questionnaire-based study conducted in a tertiary care teaching hospital in Vijayapura, India, between the months of July and August 2024.

Study setting

This study was conducted at Shri B M Patil Medical College Hospital and Research Centre, Vijayapura, India, which is a tertiary care teaching hospital that is involved in the PvPI, contributing to the ADR reporting system.

Study population and participants

The study population consisted of medical and nursing students and staff who were actively involved in patient care. Both undergraduate and postgraduate students who participated in clinical rotations and patient care as part of their training were considered medical students. All of the hospital’s practicing doctors, undergraduate or graduate, who were not students make up the medical staff. Nursing students were those enrolled in nursing programs who were participating in clinical practice, while nursing staff included all registered nurses and other nursing professionals working directly in the hospital. From this, 200 participants were selected, 100 from the medical group and 100 from the nursing group. The sample was selected based on the availability of the participants. This method was chosen for its practicality and feasibility for participants within the hospital. The informed consent was obtained electronically by using Google Forms. The participants were provided with detailed study information and took the consent form from them before proceeding to the survey.

Active participants involved in patient care (medical and nursing students and staff) were included in this study. Non-healthcare professionals and those who have taken part in recent ADR or pharmacovigilance studies were excluded from the study.

Sample size calculation

The sampling method used in this study was convenience sampling, as it allowed easy access to the targeted population [11]. The sample size [12] was calculated using the formula for estimation of population proportion: [n = Z2 × p ( 1−p ) / d2], where (n) is the required sample size, (Z) is the Z-value corresponding to the desired confidence level (1.96 for a 95% confidence level), (p) is the estimated proportion of the population expected to exhibit the characteristic of interest (knowledge/awareness about ADRs), and (d) is the margin of error or precision.

Although convenience sampling does not need a strict statistical formula for the sample size determination, this formula was used as a guideline to estimate a reasonable sample size that would provide meaningful results. Using this formula by choosing a 95% confidence level, which corresponded to a Z value of 1.96, an estimated proportion of 0.5, and a margin of error of 10%, a sample size of 96 participants was calculated for each group (medical and nursing). The sample size was rounded to 100 participants per group to account for potential dropouts and to ensure the strength of this study. Special care was taken to ensure complete data collection from every participant to minimize the risk of bias from non-responses.

Study tool

A structured and validated questionnaire consisting of 19 questions was designed to assess knowledge and awareness regarding ADRs and pharmacovigilance, and this was divided into two sections: (1) Knowledge (five questions): These questions focused on the participant's understanding of ADR, pharmacovigilance, and related concepts. (2) Awareness (14 questions): These questions focused on participants’ awareness of ADR reporting, knowledge of pharmacovigilance centers, and awareness program participation.

The questionnaire was reviewed by four academicians from the Department of Pharmacology (Shri B M Patil Medical College Hospital and Research Centre) to ensure clarity, relevance, and ease of understanding of the questionnaire. The reviewer’s response was considered for finalizing the questionnaire.

Before the survey, the questionnaire was pilot-tested on 30 medical and nursing students and staff who were excluded from the actual study, which helped to assess the reliability by calculating Cronbach’s coefficient alpha. The internal consistency of the questionnaire yielded a score of 0.72.

Data collection

The data collection was started from July to August 2024 after obtaining informed consent using online Google Forms. The questionnaire was shared with participants using email. The responses were automatically captured for further analysis. The data collection was done through an electronic distribution method and ensured that all participants had adequate time to complete the survey. The data was stored on Google Drive and was exported to Excel for analysis. The confidentiality of all data was maintained.

Data analysis

Collected data were entered into Microsoft Excel version 16.75.2 (Microsoft Corporation, Redmond, WA, USA) for analysis. Descriptive analysis was used in this study to analyze the data statistically. A comparative analysis was done to evaluate the differences between the two groups (medical and nursing) regarding their knowledge and awareness of ADR and pharmacovigilance. The results were represented using tables and graphs for better analysis.

## Results

Based on our survey to evaluate the knowledge and awareness regarding ADR and pharmacovigilance among a total of 200 medical and nursing students and staff (100 medical and 100 nursing), 98% of respondents from medical students and staff had knowledge about ADR, while 70% had knowledge about pharmacovigilance. Of nursing students and staff, 90% had knowledge about ADR, and 66% had knowledge about pharmacovigilance.

A total of 61% of nursing students and staff had knowledge about the nearest pharmacovigilance center, and 55% were knowledgeable about the ADR monitoring centers in India, compared to 41% and 51% of medical students and staff, respectively.

A total of 97% of the medical students and staff demonstrated better knowledge regarding the questions related to the importance of ADR reporting compared to 90% of nursing students and staff. Moreover, 68% of the nursing students and staff participated in the awareness programs related to ADR and pharmacovigilance compared to medical students and staff of 18%. Both medical and nursing students and staff (98% and 96%) had similar opinions that patient education and awareness will help to decrease the incidence of ADR. Table 1 summarizes the knowledge of ADR and pharmacovigilance among medical and nursing students and staff, emphasizing variations in familiarity and knowledge across both groups.

**Table 1:** Responses to the questionnaire on knowledge about ADRs and pharmacovigilance among medical and nursing students and staff ADR, adverse drug reactions

Serial number	Questions based on knowledge about ADR and pharmacovigilance	Total responses of medical students and staff (n = 100)	Total responses of nursing students and staff (n = 100)
1	Have you ever heard the term “adverse drug reaction” or side effects of drugs?	Yes – 98 (98%)	Yes – 90 (90%)
		No – 2 (2%)	No – 10 (10%)
2	Do you know the meaning of pharmacovigilance?	Yes – 70 (70%)	Yes – 66 (66%)
		No – 30 (30%)	No – 34 (34%)
3	Do you think pharmacovigilance is an aspect of pharmacoepidemiology?	Yes – 79 (79%)	Yes – 58 (58%)
		No – 21 (21%)	No – 42 (42%)
4	Do you know the nearest pharmacovigilance center located from your workplace?	Yes – 41 (41%)	Yes – 65 (65%)
		No – 59 (59%)	No – 35 (35%)
5	Do you know which organization is responsible for collecting and monitoring adverse drug reactions in India?	Yes – 51 (51%)	Yes – 55 (55%)
		No – 49 (49%)	No – 45 (45%)

**Figure 1:**
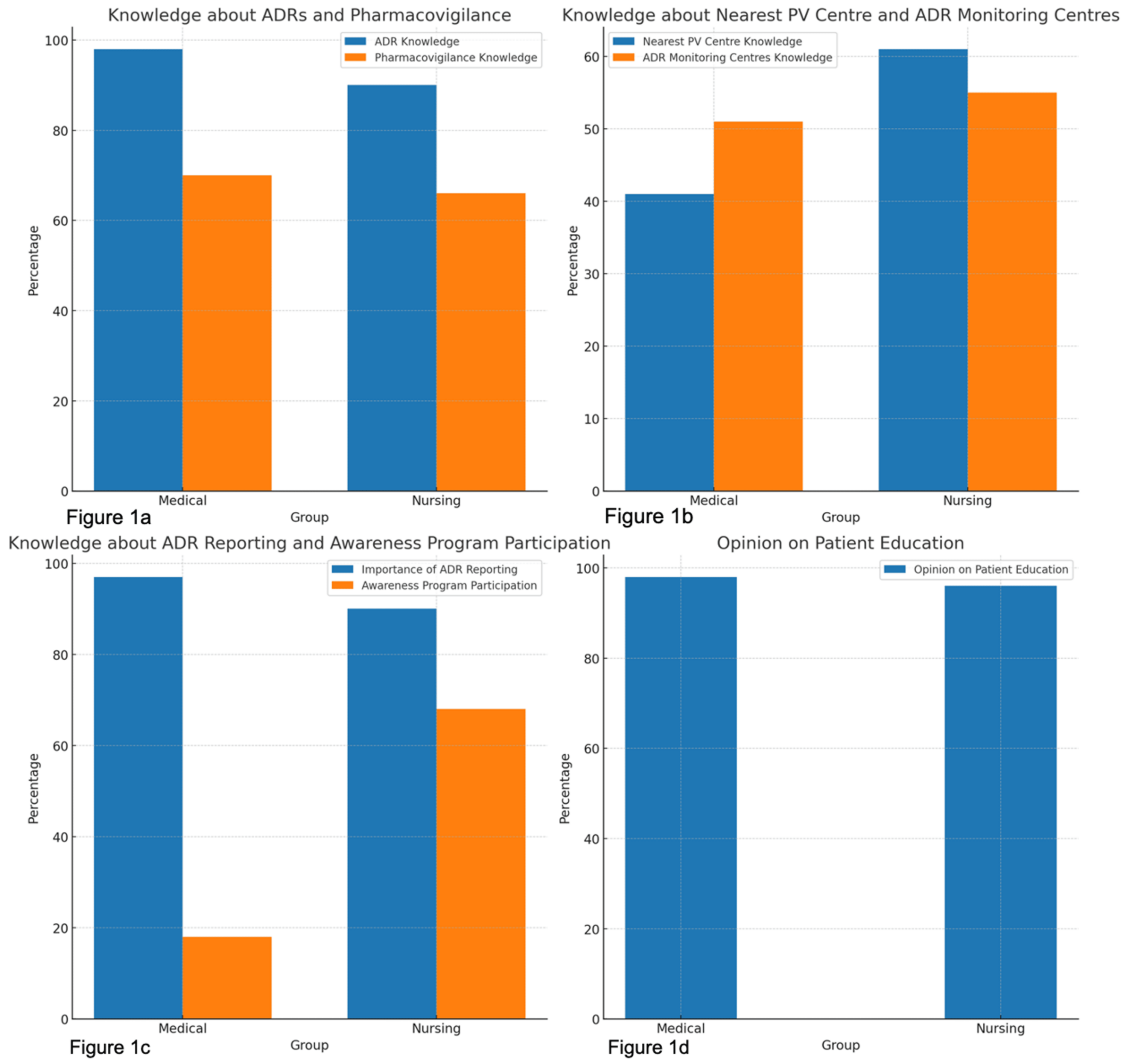
Comparison of knowledge and awareness regarding ADRs and pharmacovigilance among medical and nursing students and staff (a) This depicts the comparative analysis of knowledge about ADRs and pharmacovigilance among medical and nursing students and staff. (b) This depicts the comparative analysis of knowledge about the nearest PV centers and ADR monitoring centers among medical and nursing students and staff. (c) This depicts the comparative analysis of knowledge about ADR reporting and awareness program participation among medical and nursing students and staff. (d) This depicts the comparative analysis of opinions on the role of patient education in preventing ADR among medical and nursing students and staff. ADR, adverse drug reaction; PV, pharmacovigilance

The responses from medical and nursing students and staff regarding their awareness of ADR and pharmacovigilance, emphasizing variations in knowledge and awareness, are depicted in Table 2, and the comparative analysis of the responses to the questionnaire is represented in Figure 1a-1d.

**Table 2:** Responses to the questionnaire on awareness about ADRs and pharmacovigilance among medical and nursing students and staff ADR, adverse drug reactions

Serial number	Questions based on awareness of ADR and pharmacovigilance	Total responses of medical students and staff (n = 100)	Total responses of nursing students and staff (n = 100)
1	Do you think it’s important to report adverse drug reactions to healthcare professionals?	Yes – 97 (97%)	Yes – 90 (90%)
		No – 3 (3%)	No – 10 (10%)
2	Have you ever experienced an adverse drug reaction after taking medications?	Yes – 19 (19%)	Yes – 8 (8%)
		No – 81 (81%)	No – 92 (92%)
3	Do you report the adverse drug reaction to a healthcare professional?	Yes – 76 (76%)	Yes – 82 (82%)
		No – 24 (24%)	No – 18 (18%)
4	Have you ever been asked by a healthcare professional about any history of adverse drug reactions before starting a medication?	Yes – 72 (72%)	Yes – 78 (78%)
		No – 28 (28%)	No – 22 (22%)
5	Do you think adverse drug reactions can be prevented?	Yes – 93 (93%)	Yes – 92 (92%)
		No – 7 (7%)	No – 8 (8%)
6	Do the healthcare professionals provide information about potential adverse drug reactions before prescribing medications?	Yes – 79 (79%)	Yes – 76 (76%)
		No – 21 (21%)	No – 24 (24%)
7	Do you believe that patient education plays a role in preventing adverse drug reactions?	Yes – 98 (98%)	Yes – 96 (96%)
		No – 2 (2%)	No – 4 (4%)
8	Have you ever participated in any awareness programs or campaigns about adverse drug reactions?	Yes – 18 (18%)	Yes – 68 (68%)
		No – 82 (82%)	No – 32 (32%)
9	Do you think there is enough awareness about adverse drug reactions among the general population?	Yes – 3 (3%)	Yes – 10 (10%)
		No – 97 (97%)	No – 90 (90%)
10	Do you think adverse drug reaction reporting is only the pharmacist’s duty?	Yes – 30 (30%)	Yes – 35 (35%)
		No – 70 (70%)	No – 65 (65%)
11	Do you think reporting adverse drug reactions helps to measure the incidence of adverse drug reactions?	Yes – 91 (91%)	Yes – 94 (94%)
		No – 9 (9%)	No – 6 (6%)
12	Do you think reporting adverse drug reactions helps to identify previously unrecognized adverse drug reactions?	Yes – 90 (90%)	Yes – 93 (93%)
		No – 10 (10%)	No – 7 (7%)
13	Do you think herbal products’ adverse drug reactions should be reported?	Yes – 89 (89%)	Yes – 87 (87%)
		No – 11 (11%)	No – 13 (13%)
14	Do you know the nearest pharmacovigilance center located from your workplace?	Yes – 41 (41%)	Yes – 61 (61%)
		No – 59 (59%)	No – 39 (39%)

This study clearly shows the significance of proper education in pharmacovigilance and ADR reporting among healthcare providers. This study also emphasizes that nursing students and staff, regardless of having comparatively lesser knowledge, excel in application due to their active involvement in ADR reporting.

## Discussion

Reporting ADR is a critical aspect of the pharmacovigilance program. The spontaneous reporting system plays a significant role in documenting ADR and identifying new reactions associated with the drugs. In the present study, we aimed to evaluate the understanding of ADR and pharmacovigilance principles among medical and nursing students and staff in a tertiary care hospital.

This study shows that medical students and staff presented a higher level of expertise in answering questions on the importance of ADR reporting. The nursing students and staff participated more in the awareness programs related to ADR and pharmacovigilance. Both groups had similar opinions that patient education will make them more aware, which will ultimately help to decrease the incidence of ADR and help to find out the previously undiagnosed ADR. This study shows flexible attitudes and proper education in pharmacovigilance principles and ADR reporting, which are essential among healthcare professionals. This study will also help to form better initiatives to enhance pharmacovigilance education, fostering a culture of vigilant medication safety in healthcare.

The study conducted by Hardeep et al. [13] in a teaching hospital in North India revealed that more than 60% of clinicians were not aware of reporting ADR, which highlights the need for comprehensive education in relation to ADR reporting. Additionally, a study by Kharkar and Bowalekar [14] revealed that while medical practitioners in India generally have a positive attitude and are highly aware of ADR reporting, there is still room for improvement in reporting standards. While resident physicians and nurses showed good awareness and knowledge of ADR reporting, there was still a need for improvement in their reporting methods, as per a study by Rehan et al. [15]. A study by Upadhyaya et al. [16] found that medical students had poor knowledge regarding ADR reporting, underscoring the need to integrate ADR reporting into undergraduate education and reinforce it throughout the course and beyond.

A study by John et al. [17] emphasized the need for workshops and highlighted the importance of ADR reporting and contributions to the pharmacovigilance program in clinical practice. It revealed both the underreporting of ADRs and physicians’ willingness to undergo training.

A study by Amrita and Singh [18] found that, although doctors had good knowledge and observation of ADR, the reporting rate to ADR monitoring centers was low, and awareness of ADR reporting centers, including their contact details and the reporting form availability, was also insufficient.

A study conducted by Rajesh et al. [19] found that continuous training interventions aimed at enhancing knowledge and practice of drug safety could lead to improved ADR reporting by medical professionals. According to a study by Muraraiah et al. [20], although most medical professionals understood the importance of reporting ADRs, a lack of resources and experience made reporting difficult for them.

Another study by Palaian et al. [21] revealed that medical staff at the Manipal Teaching Hospital had improved attitudes and practices regarding ADRs, but their understanding of ADRs was still limited. The majority of respondents believed that ADR monitoring was crucial; however, few ADRs were reported because the pharmacovigilance center was not aware of them. This study highlights the need for ongoing education programs for medical practitioners.

The strength of our study lies in the comprehensive assessment of knowledge and awareness regarding ADRs and pharmacovigilance among healthcare providers. The questionnaire comprehensively covers a wide range of pharmacovigilance and ADR knowledge topics to ensure a thorough grasp of the problem. This study compares the knowledge and awareness levels of medical and nursing students and staff, providing valuable insights into the variations and facilitating the precise adaptation of more effective educational interventions.

Despite the abovementioned strengths of this study, a significant limitation is that it focuses on a single institution. The sample was not able to accurately represent every medical and nursing student, faculty, and staff in different institutions or regions, which might limit the extent to which the results can be applied. The self-reported data may also be influenced by potential biases like selection bias, sampling bias, non-response bias, and response bias, which can compromise the reliability of the data.

These findings can guide the creation of focused educational initiatives aimed at enhancing pharmacovigilance practices and improving patient care in general.

## Conclusions

This study underscores the importance of fostering flexible attitudes and providing proper education on pharmacovigilance principles and ADR reporting among healthcare professionals. Implementing effective teaching methods, such as interactive workshops and simulation-based training, can significantly improve confidence and skills in ADR reporting.

Underreporting of ADRs may stem from a lack of knowledge and insufficient awareness of the pharmacovigilance center within the hospital. The study suggests substantial potential for enhancing ongoing pharmacovigilance activities in healthcare settings. Incorporating pharmacovigilance into the curriculum is essential to building a strong culture of awareness and reporting among future healthcare professionals.
